# Sodium valproate compared to phenytoin in treatment of status epilepticus

**DOI:** 10.1002/brb3.951

**Published:** 2018-03-23

**Authors:** Mohammad Reza Amiri‐Nikpour, Surena Nazarbaghi, Parisa Eftekhari, Sedra Mohammadi, Sina Dindarian, Mahdi Bagheri, Hozan Mohammadi

**Affiliations:** ^1^ Department of Neurology Urmia University of Medical Sciences Urmia Iran; ^2^ Student Research Committee Urmia University of Medical Sciences Urmia Iran; ^3^ Student Research Committee Tabriz University of Medical Sciences Tabriz Iran

**Keywords:** phenytoin, sodium valproate, status epilepticus, tolerability

## Abstract

**Background:**

Status epilepticus (SE) is a neurological emergency which can be life‐threatening. Several medical regimens are used in order to control it. In this study, we intended to evaluate the clinical efficacy and tolerability of sodium valproate and intravenous phenytoin (IV PHT) in the control of SE.

**Methods:**

One hundred and ten consecutive patients suffering from benzodiazepine refractory SE who were referred to the emergency ward from March 2014 to March 2015 were randomly divided into two groups. The first group received intravenous sodium valproate, 30 mg/kg as loading dose and then 4–8 mg/kg every 8 hr as maintenance regimen. The second group received IV PHT 20 mg/kg as loading dose and then 1.5 mg/kg for 8 hr as maintenance therapy. All patients were monitored for vital signs every 2 hr up to 12 hr. The patients were also followed up for 7 days regarding drug response and adverse effects.

**Results:**

The administration of sodium valproate and phenytoin respectively resulted in seizure control in 43 (78.18%) and 39 (70.90%) of the patients within 7 days of drug administration (*p* = .428). Seven‐day mortality rate was similar in both groups (12.73% vs. 12.73%; *p* = .612). There was no significant difference in adverse effects between two groups.

**Conclusion:**

Sodium valproate is preferred to IV PHT for treatment and control of SE due to its higher tolerability and lower hemodynamic instability.

## 1 INTRODUCTION

Status epilepticus (SE) is one of the most common neurological emergencies, which was previously defined as long‐lasting or multiple seizures without recovery and regaining consciousness between intervals lasting for more than 30 min (Mazurkiewicz‐Bełdzińska, Szmuda, Zawadzka, & Matheisel, 2014; Reddy & Kuruba, 2013; Trinka, Höfler, Zerbs, & Brigo, 2014). This definition has been used in various studies for many years, but recently new studies proposed that for better definition, all epileptic seizures lasting more than 5 min require the same treatment as used for SE. In these patients, mechanisms for self‐termination of seizures fail. Thus, seizures can usually last for several minutes with the high possibility of recurrence (Abend et al., 2014; Reddy & Kuruba, 2013). Refractory status epilepticus (RSE) is considered as continuing seizures failing to respond to first‐ and second‐line anticonvulsant therapies (Al‐Mufti & Claassen, 2014). Long‐lasting SE can cause a wide range of complications including multiple organ derangements (such as respiratory disorders, autonomic dysfunction, rhabdomyolysis, or cardiac arrhythmias), direct damage to the brain cells due to unclear mechanisms of excessive stimulatory neurotransmitters (glutamate), and loss of inhibitory neurotransmitters gamma‐aminobutyric acid (GABA) (Mazurkiewicz‐Bełdzińska et al., 2014; Reddy & Kuruba, 2013). This life‐threatening condition can lead to high morbidity and mortality, so early diagnosis as well as treatment with effective anticonvulsants is very important, especially to prevent organ failure and metabolic disorders and stabilize cardiopulmonary function (Trinka et al., 2014). The first‐line treatment that potentiates the inhibitory responses caused by GABA‐A receptors is intravenous benzodiazepines (e.g., diazepam and lorazepam; Brophy et al., 2012). Although the initial benzodiazepines may block the seizures, their efficacy decreases with refractory exclusivity of SE and they also may cause excessive sedation and affect patient's monitoring. Therefore, it cannot be used for a long period (Al‐Mufti & Claassen, 2014; Mayer et al., 2002). Intravenous phenytoin (IV PHT) known as second‐line therapy can be used in combination with the first‐line medications to reduce recurrences. But cardiovascular and neurological adverse effects such as nervous system depression, cardiovascular collapse, or hypotension are the main disadvantages of this drug (Krishnamurthy & Drislane, 1996; Trinka et al., 2014; Wheless & Treiman, 2008; Yaffe & Lowenstein, 1993). In some cases, the seizures are so severe that they cannot be suppressed even by third‐line therapy (propofol or phenobarbital) (Wheless & Treiman, 2008). Unlike phenytoin (Abend et al., 2014; Misra, Kalita, & Bhoi, 2014; Tiamkao, Sawanyawisuth, & Chancharoen, 2013), intravenous sodium valproate (IV VPA) can be used safely against various types of SE especially for patients with cardiorespiratory impairments (Trinka et al., 2014). This nonsedating drug has high tolerability and does not cause severe hemodynamic instability (Brigo et al., 2013). In recent studies, IV VPA was more effective than phenytoin (79.0% vs. 25.0%) (Misra, Kalita, & Patel, 2006). As there are only few studies comparing the efficacy and safety of IV VPA with IV PHT, in this study we aimed to compare the efficacy of IV VPA with IV PHT in treatment of SE.

## 2 METHODS AND MATERIALS

Of all patients with epilepsy, one hundred and ten patients with benzodiazepine RSE who were referred to the Emergency Ward of Imam Khomeini Hospital, Urmia, Iran, from March 2014 to March 2015 were included in the study. All participants had SE, defined as a continuous generalized convulsive seizure lasting greater than 5 min or two or more discrete seizures during which the patient had not returned to baseline consciousness (Lowenstein, Bleck, & Macdonald, 1999). Pregnant women, patients younger than 18 years old, patients with history of liver diseases, patients requiring emergency neurological invasive interventions, and finally patients suffering from hypotension, pancreatitis, congestive heart failure, cardiac arrhythmias, postanoxic SE, nonepileptic seizures, or sensitivity to phenytoin or sodium valproate were all excluded from the study.

Baseline characteristics, medical history, and laboratory parameters were retrieved from medical files recorded in hospital. The participants were randomly divided into two groups using block randomization method. The first group received intravenous sodium valproate (Depakine; Sanofi‐Aventis), 30 mg/kg as the loading dose and then 4–8 mg/kg every 8 hr as maintenance regimen. The second group received intravenous phenytoin (Hydantoic; Caspian Tamin), 20 mg/kg as loading dose and then 1.5 mg/kg every 8 hr as maintenance therapy. Before being labeled as RSE, all patients had been treated by IV diazepam in doses of 0.2 mg/kg at 2 mg/min up to a maximum 20 mg. The patients were monitored for vital signs including blood pressure, heart rate and respiratory rate every 2 hr up to 12 hr. The patients were also followed up for 7 days regarding drug response and adverse effects. Furthermore, all participants were tested for complete blood count, liver enzymes, serum electrolytes, blood sugar, serum urea, and creatinine as well as cerebrospinal fluid (CSF) analysis and magnetic resonance imaging (MRI) for determining etiologies of seizures.

Results were presented as mean ± standard deviation (*SD*) for quantitative variables and were summarized by absolute frequencies and percentages for categorical variables. Categorical variables were compared using chi‐square test or Fisher's exact test when more than 20% of cells with expected count of <5 were observed. Continuous variables were compared using one‐way analysis of *t* test and/or nonparametric Mann–Whitney test whenever the data did not appear to have normal distribution.

All experiments were reviewed by the local ethical committee of Urmia University of Medical Sciences, Urmia, Iran, and were approved as a student thesis with the approval decision number of 90‐02‐32‐655 in October 2013. The experiments were also in complete accordance with 1964 Helsinki declaration and its later amendments or comparable ethical standards.

## 3 RESULTS

### 3.1 Demographic factors

Mean age of the patients in sodium valproate and phenytoin groups was 42.16 ± 15.94 and 43.69 ± 17.60, respectively. In sodium valproate group, there were 24 males and 31 females. Also, there were 27 males and 28 females in phenytoin group.

### 3.2 Etiologies

In both groups, the most common etiology of SE was drug withdrawal with, respectively, 19 (34.54%) and 18 (32.74%) patients in sodium valproate and phenytoin groups. The primary generalized seizure was the second most common etiology in both groups with 10 (18.20%) patients in each. The third most common etiology of SE was brain stroke including ischemic and hemorrhagic brain strokes with eight (14.54%) cases in sodium valproate group and seven (12.72%) cases in phenytoin group. Other etiologies explained in Table 1 include acute, progressive, and remote etiologies such as infections, tumors.

**Table 1:** Etiologies of status epilepticus in the patients received VPA or PHT

Etiology	VPA	PHT
Drug discontinuation	19 (34.54)	18 (32.74)
Herpetic encephalitis	2 (3.63)	3 (5.45)
Hypocalcemia	2 (3.63)	1 (1.82)
Brain stroke	8 (14.54)	7 (12.72)
Paraneoplastic encephalitis	1 (1.82)	0 (0)
Metastatic brain tumors	6 (10.91)	3 (5.45)
Uremia	1 (1.82)	2 (3.63)
Primary generalized seizures	10 (18.20)	10 (18.20)
Eclampsia	1 (1.82)	0 (0)
Tramadol abuse	3 (5.45)	4 (7.28)
Cerebral tuberculosis	1 (1.82)	1 (1.82)
Sturge–Weber syndrome	1 (1.82)	0 (0)
Bacterial meningitis	0 (0)	2 (3.63)
Cerebral venous thrombosis	0 (0)	2 (3.63)
Primary brain tumor	0 (0)	2 (3.63)
Total	55 (100)	55 (100)

PHT, phenytoin; VPA, sodium valproate.

### 3.3 MRI findings

Brain MRI results were available for all of the patients. Of 110 patients, 60 (54.5%) showed abnormal brain MRI (Table 2).

**Table 2:** MRI findings in the patients treated with VPA or PHT

MRI findings	VPA (*n* = 55)	PHT (*n* = 55)
Normal	24 (43.6)	26 (47.3)
Encephalomalacia due to head trauma	2 (3.6)	1 (1.8)
Bilateral temporal lesions (Herpes simplex encephalitis)	2 (3.6)	3 (5.5)
Basal ganglia calcification	2 (3.6)	1 (1.8)
Brian hemorrhage	1 (1.8)	1 (1.8)
Lobar hemorrhage	1 (1.8)	0
Subarachnoid hemorrhage	0	1 (1.8)
Small vessel disease	4 (7.3)	3 (5.5)
Cavernous angioma	1 (1.8)	0
Occipital lobe lesions	1 (1.8)	0
Multiple white matter lesions	1 (1.8)	0
Periventricular lesions	1 (1.8)	0
Brain metastasis	6 (10.9)	3 (5.5)
Diffuse brain ischemia	7 (12.7)	5 (9.1)
Primary brain tumor	0	3 (5.5)
Arachnoid cysts	1 (1.8)	2 (3.6)
Venous sinus thrombosis	0	2 (3.6)
Meningeal involvement	0	1 (1.8)
Basilar leptomeningeal enhancement due to tuberculous meningitis	0	1 (1.8)
Congenital brain malformation	1 (1.8)	1 (1.8)
Brain granulomas	0	1 (1.8)

MRI, magnetic resonance imaging; PHT, phenytoin; VPA, sodium valproate.

Most common brain abnormalities in VPA group included the following:

1. Diffuse brain ischemia: Seven (12.7%) patients showed brain ischemia (i.e., due to head trauma).
2. Brain metastasis: Six (10.9%) patients showed brain metastasis.
3. Small vessel disease: Four (7.3%) cases were presented with small vessel defects in MRI, which were commonly caused by diabetes mellitus (DM) or hypertension (HTN).

Most common brain abnormalities in PHT group encompassed the following:

1. Diffuse brain ischemia in five (9.1%) patients.
2. Bilateral temporal lesions (due to herpes simplex encephalitis), small vessel disease, brain metastasis, and primary brain tumor each in three cases (5.5%).

### 3.4 Lumbar puncture (LP) findings:

Of 110 patients, 63 underwent LP and other 47 patients refused to give consent for that. Fifty‐seven (51.8%) of these patients had normal LP. Three patients in VPA group and four patients in PHT group had lymphocytic pleocytosis in their LP findings. Furthermore, one patient in VPA group and two patients in PHT group had either high protein or low glucose in their LP findings (Table 3).

**Table 3:** LP findings in the patients treated with VPA or PHT

LP findings	VPA (*n* = 55)	PHT (*n* = 55)
Normal	34 (61.8)	19 (34.5)
Lymphocytic pleocytosis	3 (5.5)	4 (7.3)
High protein or low glucose	1 (1.8)	2 (3.6)
No lumbar puncture	17 (30.9)	30 (54.5)

LP, lumbar puncture; PHT, phenytoin; VPA, sodium valproate.

### 3.5 Seizure control and outcomes

Administration of VPA and PHT could control seizures in, respectively, 43 (78.18%) and 39 (70.90%) patients within 7 days of administration (*p* = .428). In 12 patients with uncontrolled SE in VPA group, seven died and other five patients were refractory to VPA. Also in PHT group, in 16 patients with uncontrolled SE, seven patients died and other nine patients were refractory to PHT. Seven‐day mortality rate was 12.7% in both groups (*p* = .61; Table 4). None of the patients in VPA group and three (5.45%) patients in PHT group developed hypotension. Bradycardia was found in none of the patients in VPA group and two (3.64%) patients in PHT group. Also, one (1.82%) patient in VPA group and no patients in PHT group developed bradypnea. Finally, elevated liver enzymes were observed in 5.45% of patients in VPA group and 1.82% of the patients in PHT group (Table 5).

**Table 4:** Seven‐day outcome in the patients treated with VPA or PHT

Outcomes	VPA (*n* = 55)	PHT (*n* = 55)	*p*‐value
SE controlled	43 (78.18)	39 (70.90)	.428
Mortality	7 (12.73)	7 (12.73)	.612

PHT, phenytoin; SE, status epilepticus; VPA, sodium valproate.

**Table 5:** Adverse effects of treatment with VPA or PHT

Adverse effect	VPA (*n* = 55)	PHT (*n* = 55)	*p*‐value
Hypotension	0	3	.213
Bradycardia	0	2	.358
Bradypnea	1	0	.558
Raised liver enzymes	3	1	.447

PHT, phenytoin; VPA, sodium valproate.

## 4 DISCUSSION

Regimens including sodium valproate and phenytoin are two common regimens for treatment and control of SE. In this study, we aimed to compare both drug efficacy and tolerability of these regimens in benzodiazepine refractory SE patients. Despite administration of either drug for patients with SE, preference for one drug over another has remained uncertain. The uncertainty is due to partial tolerability and potential adverse effects of each drug. The results of our study demonstrated high efficacy of each drug in the control of SE in a 7‐day treatment period; however, it seems that sodium valproate was more tolerable than phenytoin especially with regard to early changes in blood pressure in case of phenytoin administration. On the other hand, despite relatively high 7‐day mortality rate in patients treated with either medication in our study, using sodium valproate has resulted in lower rate of hemodynamic instability leading to a more favorable long‐term outcome regarding lower late mortality and morbidity.

Recently, the comparison of efficacy and tolerability of these two drugs has gained more attention. In a study by Misra et al. (2006), higher drug efficacy was revealed following the administration of sodium valproate because of higher seizure abortion when compared with intravenous phenytoin (65.7% vs. 42.0%). The adverse effects were similar in each group in that study. In a study carried out by Chitsaz, Mehvari, Salari, Gholami, and Najafi (2013), there was no difference in clinical efficacy between the two treatment protocols, but lower clinical adverse effects were shown following prescription of sodium valproate which made it more preferable over treatment with phenytoin. Tiamkao et al. (2013) also showed complete similarity in clinical efficacy between sodium valproate and phenytoin groups. Interestingly, in their study, no cardiovascular adverse event was observed in each treatment group. Gilad et al. (2008) also found similar efficacy in the groups treated with sodium valproate and phenytoin (87.5% vs. 88.0%) with considerably lower adverse effects in sodium valproate group (0.0% vs. 12.0%). This indicates higher tolerability of sodium valproate in comparison with phenytoin. Agarwal et al. (2007) also demonstrated similar efficacy of sodium valproate and phenytoin in controlling seizure and preventing its recurrence. Thus, it can be concluded that considering the findings of previous studies and our study, the therapeutic effects of sodium valproate are completely similar to intravenous phenytoin, but the adverse effects may be lower after administration of sodium valproate.

An important point in treatment of patients with SE is a relatively high mortality rate in spite of using appropriate antiepileptic drugs (AEDs). In our study, 7‐day mortality rate in both treatment groups was 12.7% with no difference between two groups. Despite high early mortality reported in some studies (Boggs, 2004; Koubeissi & Alshekhlee, 2007), the mortality rate due to SE remains confusing due to differences in population demographics, follow‐up time, definition of SE, and timing of death. Thus, a wide range (2.5%–43.0%) of 30‐day mortality rate has been observed in the previous studies (DeLorenzo et al., 1999; Logroscino et al., 2002). Totally, considering 7‐day mortality rate in our study, we may also expect high 30‐day mortality rate which can be either due to no response to drug in control of seizure or hemodynamic instabilities after administration of AEDs.

In this study, due to low number of patients in each etiology group, we were not able to assess the correlation between etiology of SE and outcome of treatment with either PHT or VPA. In the study of Tiamkao et al. (2013), no statistically significant correlation was observed between etiology of SE and its control using the drugs. On the other hand, Neligan and Shorvon (2011) suggested that control of SE can be dependent on its etiology. They stated that some etiologies such as AED withdrawal‐induced SE can be controlled easier than some other etiologies. Further studies are recommended to be carried out in order to assess whether the etiology is important in the control of SE using AEDs.

In the current study, we observed relatively fewer adverse effects in sodium valproate group compared with phenytoin group. Our results agree with results of the study carried out by Tiamkao et al. (2013). In their study, patients treated with VPA had better functional outcomes at discharge in comparison with patients treated by PHT. Also, Liu, Wu, Chen, Ma, and Su (2012) and Chitsaz et al. (2013) stated low clinical complications in VPA group. Consequently, VPA seems to be safer than PHT in controlling SE.

In conclusion, considering the similar clinical efficacy of sodium valproate and IV PHT in treatment of SE, sodium valproate is preferred to phenytoin because of lower hemodynamic instability. Due to limitations of our trial including small sample size, short‐term follow‐up, and ignoring some other factors such as duration of hospital stay, advantages of sodium valproate over phenytoin should be reassessed in further clinical trials.

## CONFLICT OF INTEREST

None declared.
